# FurC (PerR) contributes to the regulation of peptidoglycan remodeling and intercellular molecular transfer in the cyanobacterium *Anabaena* sp. strain PCC 7120

**DOI:** 10.1128/mbio.03231-23

**Published:** 2024-02-09

**Authors:** Cristina Sarasa-Buisan, Mercedes Nieves-Morión, Sergio Arévalo, Richard F. Helm, Emma Sevilla, Ignacio Luque, María F. Fillat

**Affiliations:** 1 Departamento de Bioquímica y Biología Molecular y Celular, Facultad de Ciencias e Instituto de Biocomputación y Física de Sistemas Complejos. Universidad de Zaragoza, Zaragoza, Spain; 2 Instituto de Bioquímica Vegetal y Fotosíntesis, CSIC and Universidad de Sevilla, Sevilla, Spain; 3 Department of Biochemistry, Virginia Tech, Blacksburg, Virginia, USA; Duke University School of Medicine, Durham, North Carolina, USA

**Keywords:** cyanobacteria, FurC (PerR), exoproteome, peptidoglycan, nanopores

## Abstract

Microbial extracellular proteins and metabolites provide valuable information concerning how microbes adapt to changing environments. In cyanobacteria, dynamic acclimation strategies involve a variety of regulatory mechanisms, being ferric uptake regulator proteins as key players in this process. In the nitrogen-fixing cyanobacterium *Anabaena* sp. strain PCC 7120, FurC (PerR) is a global regulator that modulates the peroxide response and several genes involved in photosynthesis and nitrogen metabolism. To investigate the possible role of FurC in shaping the extracellular environment of *Anabaena*, the analysis of the extracellular metabolites and proteins of a *furC*-overexpressing variant was compared to that of the wild-type strain. There were 96 differentially abundant proteins, 78 of which were found for the first time in the extracellular fraction of *Anabaena*. While these proteins belong to different functional categories, most of them are predicted to be secreted or have a peripheral location. Several stress-related proteins, including PrxA, flavodoxin, and the Dps homolog All1173, accumulated in the exoproteome of *furC*-overexpressing cells, while decreased levels of FurA and a subset of membrane proteins, including several export proteins and *amiC* gene products, responsible for nanopore formation, were detected. Direct repression by FurC of some of those genes, including *amiC1* and *amiC2,* could account for odd septal nanopore formation and impaired intercellular molecular transfer observed in the *furC*-overexpressing variant. Assessment of the exometabolome from both strains revealed the release of two peptidoglycan fragments in *furC*-overexpressing cells, namely 1,6-anhydro-N-acetyl-β-D-muramic acid (anhydroMurNAc) and its associated disaccharide (β-D-GlcNAc-(1-4)-anhydroMurNAc), suggesting alterations in peptidoglycan breakdown and recycling.

**IMPORTANCE:**

Cyanobacteria are ubiquitous photosynthetic prokaryotes that can adapt to environmental stresses by modulating their extracellular contents. Measurements of the organization and composition of the extracellular milieu provide useful information about cyanobacterial adaptive processes, which can potentially lead to biomimetic approaches to stabilizing biological systems to adverse conditions. *Anabaena* sp. strain PCC 7120 is a multicellular, nitrogen-fixing cyanobacterium whose intercellular molecular exchange is mediated by septal junctions that traverse the septal peptidoglycan through nanopores. FurC (PerR) is an essential transcriptional regulator in *Anabaena*, which modulates the response to several stresses. Here, we show that *furC*-overexpressing cells result in a modified exoproteome and the release of peptidoglycan fragments. Phenotypically, important alterations in nanopore formation and cell-to-cell communication were observed. Our results expand the roles of FurC to the modulation of cell-wall biogenesis and recycling, as well as in intercellular molecular transfer.

## INTRODUCTION

Cyanobacteria are photosynthetic prokaryotes whose outstanding metabolic plasticity allows them to populate a wide range of habitats. As cyanobacteria are exposed to dynamic environmental conditions, they have developed sophisticated defense and detoxification systems to ameliorate the consequences of stress-induced reactive oxygen species (ROS). While the dynamics of cyanobacterial intracellular processes related to stress responses has been studied intensively (1–12), there are significant gaps in our understanding of the stresses managed in the extracellular space. In addition to a variety of exometabolites, cyanobacterial extracellular milieu is rich in proteins, whether they are actively secreted or not (13–18). As part of the exoproteome, cyanobacteria release outer membrane vesicles, which could be involved in cell-to-cell communication, nutrient uptake, and the management of several environmental stresses (19–21). At present, only a few comparative analyses of cyanobacterial exoproteomes performed under nutritional or other stress conditions are available (13, 14, 16, 22, 23). The presence of several metal-related proteins, as well as others normally involved in the intracellular oxidative stress response, such as superoxide dismutases, catalases, and rubrerytrin, has been described in the exoproteome of different cyanobacteria (13, 14, 22). Actually, previous information about the exoproteome of the organism under study, the filamentous nitrogen-fixing cyanobacterium *Anabaena* sp. strain PCC 7120 (herein named *Anabaena*), revealed not only the presence of such proteins but also their enzymatic activities, detecting catalase and superoxide dismutase activities in the isolated culture supernatants (13). Additionally, several analyses of the *Anabaena* and *Nostoc* exoproteomes also showed perturbations related to nutrient availability (i.e., combined nitrogen) and changes in the composition and organization of the extracellular matrix influencing cellular communication with the environment (13, 14).

In the absence of combined nitrogen, specific cells in *Anabaena* filaments differentiate into heterocysts, in which atmospheric N_2_ is reduced by nitrogenase (24, 25). To preserve oxygen-sensitive nitrogenase activity, heterocysts lack oxygen-producing photosystem II and are encased in a multilayered envelope containing heterocyst-specific polysaccharide and glycolipid layers. Filamentous cyanobacteria are, therefore, multicellular organisms with programmed cell division. Hence, environmental stresses affect not only filament relationships with the extracellular milieu but also conditions such as intercellular communication, differentiation, and mass transfer processes (26). Septal junctions (SJs) are gap junction analogs in cyanobacteria that connect the cytoplasms of adjacent cells by crossing through discrete perforations in the peptidoglycan (PG) layer termed nanopores (27). Septal junction closure is triggered by the loss of membrane potential, as well as by different stresses, due to a structural rearrangement, which induces the closure of SJs gating cell connection and molecular exchange, resulting in reversible loss of communication and mass transfer between cells (28). However, until present, the regulation of these mechanisms is not completely understood.

Ferric uptake regulator (FUR) proteins are prokaryotic metalloregulators that control a large number of genes involved in different stress responses. In *Anabaena*, the FUR family consists of three paralogs, namely FurA (Fur), FurB (Zur), and FurC (PerR) (29). The *Anabaena* peroxide response regulator FurC is a global regulator whose regulon expands beyond the control of the oxidative stress response and includes genes involved in heterocyst differentiation, phycobilisome assembly, and cell division, among other functions (30, 31). Since FurC is an important environmental regulator in filamentous cyanobacteria, we hypothesized that *furC* overexpression would result in changes in the excreted proteins and metabolites that would provide insight into *Anabaena* adaptive processes. Proteomic analyses revealed a reduction in levels of AmiC and other proteins involved in cell-wall biogenesis in the extracellular milieu of the *furC*-overexpressing strain. This led us to evaluate nanopore formation and intercellular communication in this variant. Our results highlight the contribution of this transcriptional regulator in the modulation of general cell-wall biogenesis and recycling processes, as well as in the intercellular transfer along the filament.

## RESULTS

### Overexpression of *furC* highly influences the extracellular proteomic profile of *Anabaena*

**Fig 1:**
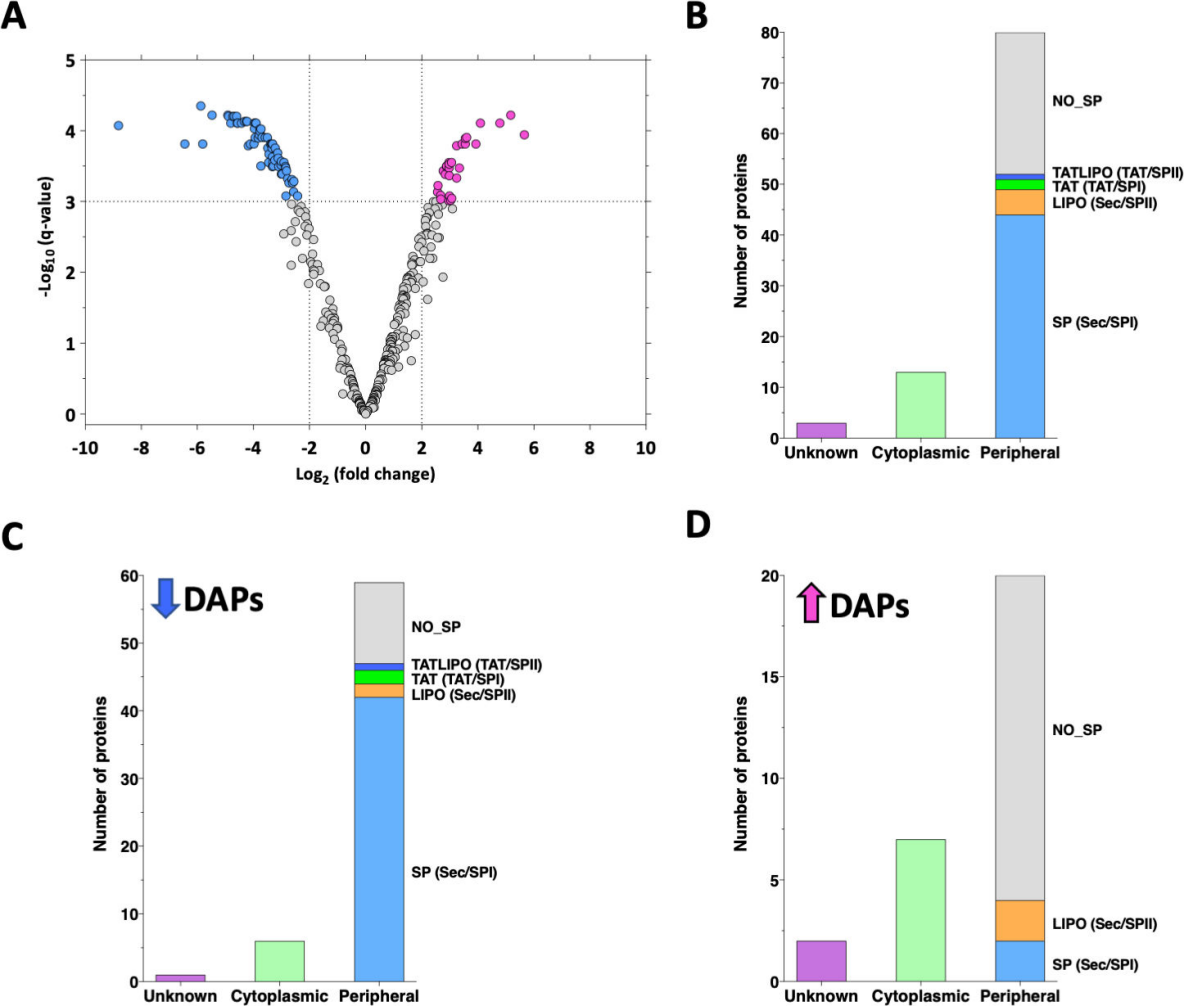
An overview of quantitative proteomic results in EB2770FurC and WT exoproteomes. (**A**) Volcano plot displaying DAPs in the exoproteome of EB2770FurC and WT. Proteins are ranked in a volcano plot according to their statistical false discovery rate (FDR)-adjusted *P*-value “*q*-value” (*y*-axis) as –log_10_ and their relative abundance ratio (log_2_ fold change [EB2770FurC vs WT]). The cutoffs for significant changes are a fold change of −2 or less or ≥2 and a *q*-value ≤ 0.001 (−Log_10_*q*-value ≥ 3). Blue spots show the proteins significantly underrepresented in the exoproteome of EB2770FurC, and pink spots show the proteins significantly overrepresented. (**B, C, and D**) Prediction of subcellular localization of the (**B**) total DAPs, (**C**) underrepresented DAPs, or (**D**) overrepresented DAPs in EB2770FurC exoproteome. The presence and type of signal peptide predicted by SignalP6 are shown for proteins with peripheral localization. NO SP, no signal peptide; SP (Sec/SPI), “standard” secretory signal peptides transported by the Sec translocon and cleaved by signal peptidase I (*Lep*); LIPO (Sec/SPII), lipoprotein signal peptides transported by the Sec translocon and cleaved by signal peptidase II (*Lsp*); TAT (Tat/SPI), Tat signal peptides transported by the Tat translocon and cleaved by signal peptidase I (*Lep*); TATLIPO (Tat/SPII), Tat lipoprotein signal peptides transported by the Tat translocon and cleaved by signal peptidase II (*Lsp*).

The exoproteome fractions obtained from cultures of two biological replicates of a *furC*- overexpressing strain, EB2770FurC (31), and wild type (WT) *Anabaena* were analyzed by sequential window acquisition of all theoretical mass spectra (SWATH-MS) to determine the differences in released proteins between both strains. There were 96 proteins significantly under- or overrepresented (*P* value ≤ 0.001; log2 fold change ≤ −2 or ≥ 2) in the exoproteome fraction of EB2770FurC with respect to that of the WT strain (hereafter differentially abundant proteins [DAPs]; Fig. 1; Table S2). From the 96 total DAPs, 18 had been previously identified in the exoproteome fraction of *Anabaena*, whereas 78 correspond to proteins identified *de novo* in the extracellular fraction of this cyanobacterium (Table S2). As shown in Fig. 1A, the number of DAPs displaying lower levels in the EB2770FurC exoproteome vs WT *Anabaena* was higher (67 DAPs) than the number of proteins that showed increased abundance (29 DAPs; Fig. 1A).

The predictions for subcellular localization, presence of signal peptides, and putative secretion pathways indicate that the exoproteome of EB2770FurC is highly enriched in “peripheral” proteins (either predicted to be secreted, periplasmic, or located at the periphery of the lipid bilayer and likely loosely bound to cell membranes). Fig. 1B shows that among 96 total DAPs, 80 (83%) were predicted to be “peripheral” (to be secreted and/or have extracellular/peripheral location), containing most of them a predicted Sec/SpI signal peptide. However, when higher and lower abundant DAPs were analyzed independently (Fig. 1C and D), clear differences in their distribution patterns were observed. Most underrepresented DAPs in the EB2770FurC exoproteome were predicted to be peripheral (90%), whereas distribution of DAPs present at higher relative abundance levels is more diversified, having predicted peripheral (69%), cytoplasmic (24%), or unknown (7%) localizations (Fig. 1C). Interestingly, most of the peripheral less abundant proteins contained the Sec/SPI signal peptide type predicted to be translocated by the general secretory (sec) pathway, while higher abundant proteins showed a clear enrichment of nonclassical secretion pathway proteins (Fig. 1D).

**Fig 2:**
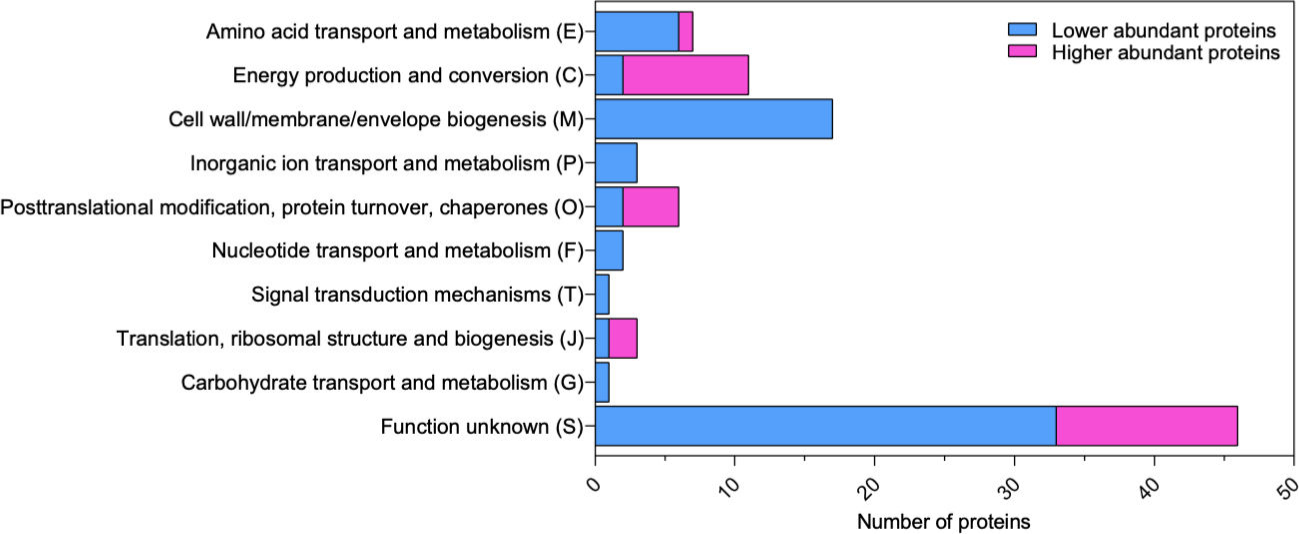
Functional classification of total DAPs in the exoproteome of the *furC*-overexpressing strain EB2770FurC relative to that of *Anabaena* sp. strain PCC 7120. Functional categorization according to COG classification of lower abundance (blue) and higher abundance (pink) proteins.

The over- and underrepresented proteins in the exoproteome of the *furC*-overexpressing strain were separately sorted into different functional categories according to the Cluster of Orthologous Groups (COG) classification (Fig. 2; Table S2). While 52 proteins were classified into functional categories, the remaining 44 DAPs corresponded to hypothetical or poorly characterized proteins. As seen in Fig. 2, under- and overrepresented proteins in the EB2770FurC exoproteome differed in their functional profiles. Most of the proteins with a known function that presented higher abundance in EB2770FurC exoproteome belonged to the functional categories of “energy production and conversion and translation (nine DAPs)” and “posttranslational modification, turnover, and chaperones (four DAPs),” whereas in the underrepresented proteins of EB2770FurC, the cell-wall-related functional category (17 DAPs) is the most prominent (Fig. 2; Table S2). This group includes several porin-like proteins, namely the major outer membrane proteins Alr0834 (OprB-1), Alr4741, Alr7614, Alr4893, All4499, Alr4550, and Alr2269 (Omp85) (13, 32, 33); the outer membrane TolC transporter homolog HgdD (Alr2887) (34); the N-acetylmuramyl-L-alanine amidases AmiC1, AmiC2, and All4999 (35, 36); and LptA (Alr4067), a protein of the lipopolysaccharide export system to the outer membrane (37), among others. Finally, it is noteworthy the strong decrease in the two-component hybrid sensor and regulator All1178 (−451.68-fold), whose function is unknown, and the FurA paralog (38) (−87.48-fold).

Outstanding DAPs with known functions overrepresented in the supernatant of EB2770FurC cultures were related to detoxification and protection against ROS, namely, the peroxiredoxin PrxA (11.69-fold higher) and the DNA-binding starvation-inducible protein All1173 (7.84-fold) previously detected in the exoproteome of *Anabaena* (13, 39), as well as the chaperonins GroES (7.86-fold) and GroL1 (7.38-fold) detected in this fraction here for the first time. Furthermore, the electron transport proteins ferredoxin-NADP^+^ reductase (PetH; 6.38-fold) and flavodoxin (IsiB; 5.89-fold; Table S2), whose presence was previously reported in the exoproteome fraction of *Anabaena* (13, 39), were also more abundant in the EB2770FurC exoproteome.

### The *furC-*overexpressing strain releases PG fragments

**Fig 3:**
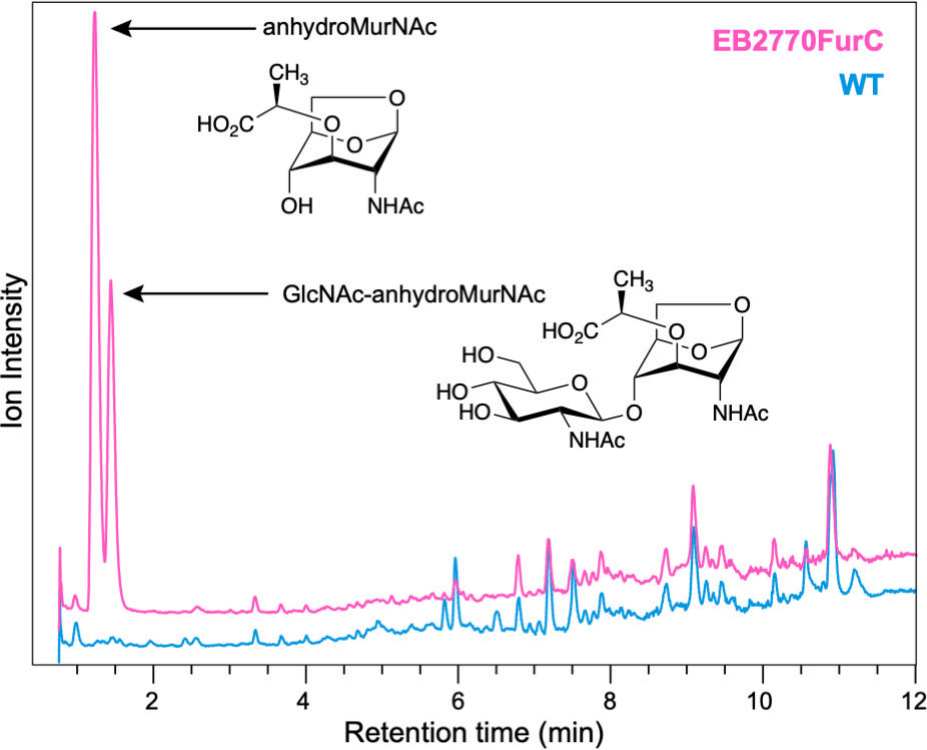
Accumulation of specific cell wall fragments in cell-free media of *Anabaena* sp. strain PCC7120 and EB2770FurC cultures. Filtrated supernatants of the same cultures that were used for exoproteome profile determination were analyzed by LC-MS for the exometabolome profiling (WT, blue; EB2770FurC, pink). Structures were confirmed with authentic standards (for MS/MS, see Fig. S1).

Untargeted small-molecule analysis (liquid chromatography mass spectrometry [LCMS]-based) of the filtered supernatants from *Anabaena* sp. PCC7120 and EB2770FurC cells revealed that *furC*-overexpressing cells released two PG fragments, namely, 1,6-anhydro-*N*-acetylmuramic acid (anhMurNAc) and its associated β-(1-4)-linked disaccharide (GlcNAc-anhMurNAc). Both of these compounds were below the limit of detection in the WT strain media (Fig. 3). Anhydro-based PG fragments that do not contain a stem peptide result from the processing of the “reducing ends” of PG, indicating that FurC expression is associated with PG recycling processes (40, 41).

### Integrity of the cell envelope is affected in the *furC*-overexpressing strain

**Fig 4:**
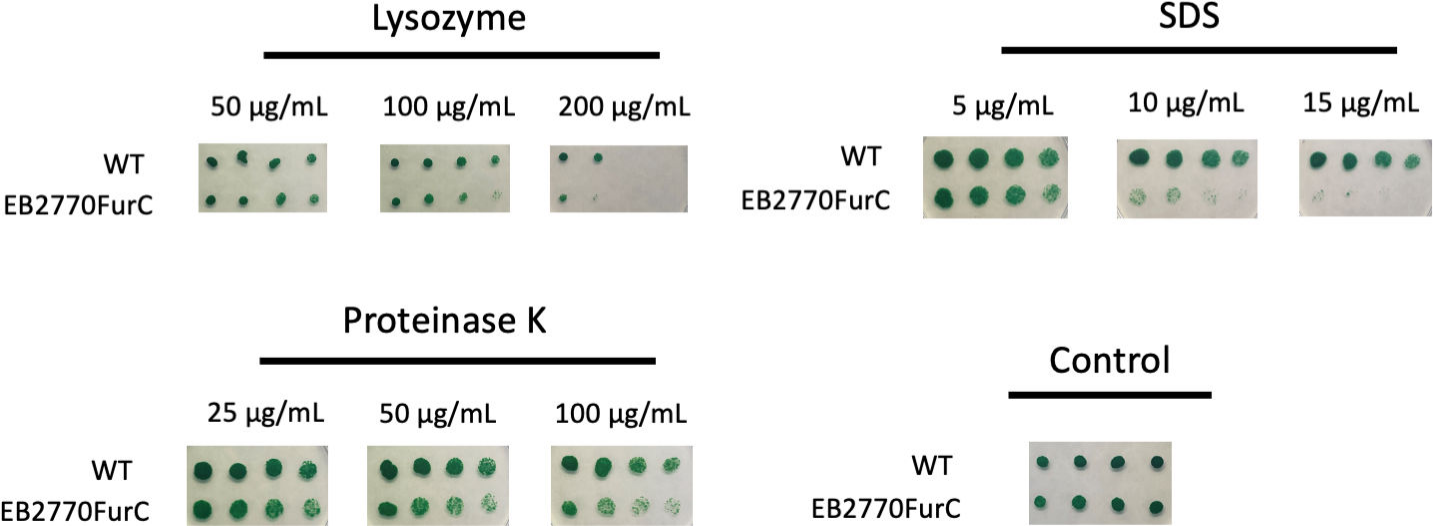
Assessment of the outer membrane integrity in EB2770FurC cells. Representative images of the outer membrane integrity assays of EB2770FurC cells. 5 µL of serial twofold dilutions of WT *Anabaena* sp. strain PCC7120 and the *furC-*overexpressing strain EB2770FurC at OD_750_ = 1.0 were spotted onto BG11C plates containing the indicated concentrations of the harmful compounds. Images were taken after 6 days of growth.

In order to analyze the potential effects of *furC* overexpression on the integrity of the cell envelope, the effect of several harmful compounds that may affect the growth of strains with a compromised envelope was tested. As illustrated in Fig. 4, the addition of lysozyme, which catalyzes peptidoglycan hydrolysis, slightly hampered the growth of EB2770FurC cells, as did the addition of proteinase K. However, the most pronounced effect was observed when adding SDS (Fig. 4; Fig.S2).

### FurC directly regulates the expression of genes involved in cell-wall biogenesis and nanopore formation

**Fig 5:**
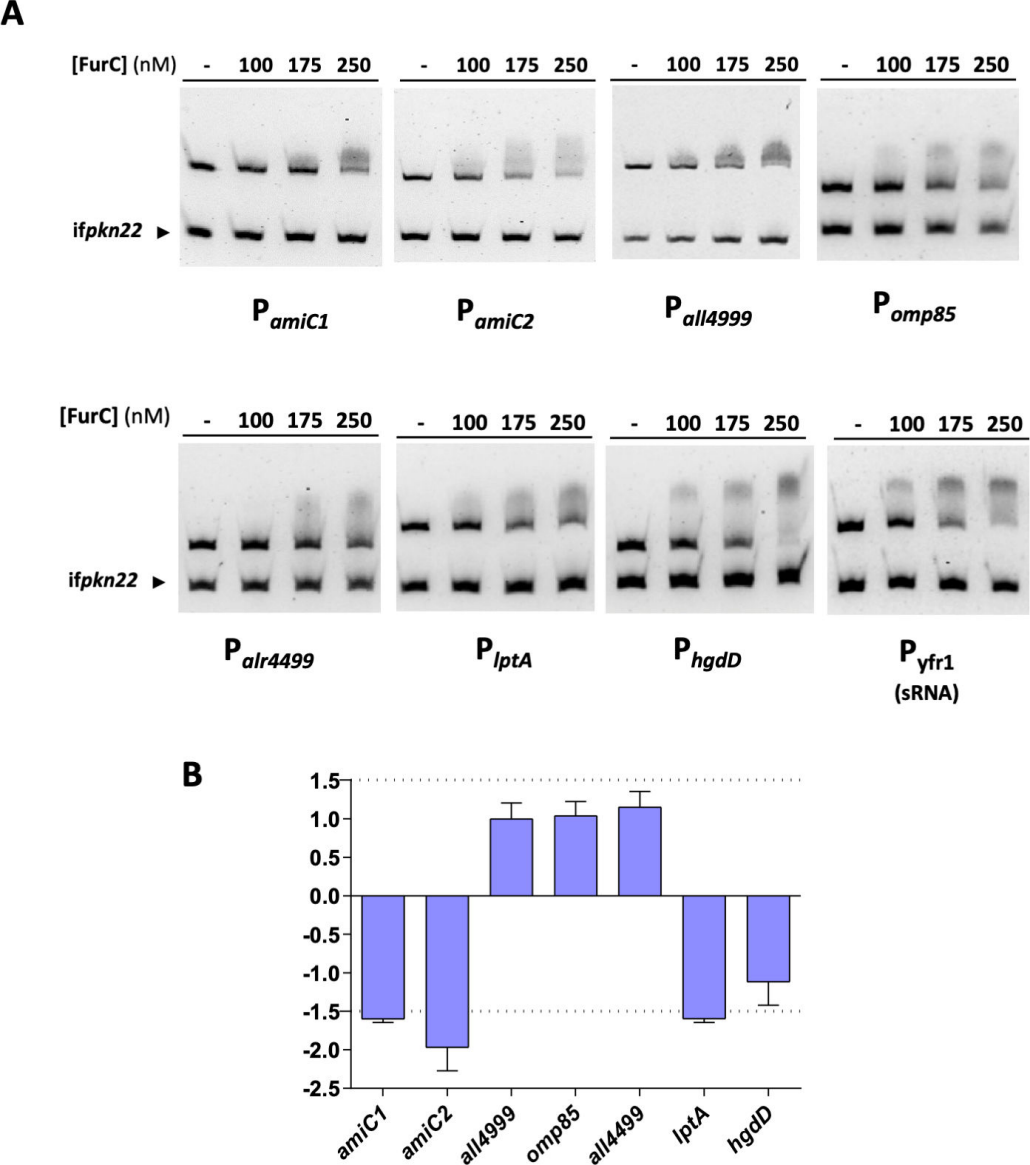
Evaluation of FurC direct regulation of genes coding for cell-wall and PG remodeling-related proteins showing lower levels in the exoproteome of a *furC*-overexpressing strain. (**A**) EMSAs were performed to test the *in vitro* interaction between FurC and the promoter regions of the selected genes. All assays were performed with DNA fragments free or incubated along with the indicated increasing concentrations of FurC (nM), separated in 6% PAGE gels. The internal fragment of gene *pkn22* (if*pkn22*) was used as nonspecific competitor DNA. (**B**) Relative transcription of *amiC1*, *amiC2*, *all4999*, *omp85*, *all4499, lptA*, and *hgdD* genes was determined by real-time RT-PCR. Values are expressed as fold change and correspond to the average of three biological and three technical replicates. The SD is indicated. The threshold of fold change ±1.5 is shown in dotted lines.

Potential direct repression by FurC of several underrepresented DAPs related to cell-wall biogenesis in the EB2770FurC exoproteome was assessed by electrophoretic mobility shift assay (EMSAs; Table 1; Fig. 5A). FurC bound to the promoter regions of amidase-encoding genes such as *amiC1*, *amiC2*, and *all4999*; the lipopolysaccharide export system genes such as *lptA*; and porin-encoding genes such as *omp85* and *alr4499*, and *hgdD*. The influence of FurC overexpression on the transcriptional levels of these genes was further analyzed by real-time reverse transcription-PCR (RT-PCR) comparing the relative changes in mRNA levels between EB2770FurC and the WT strain. Fig. 5B shows that transcription of both murein amidases *amiC1* and *amiC2*, as well as of *lptA*, was downregulated in the EB2270FurC strain (–1.60-, –1.97-, and −1.60-fold, respectively), indicating that FurC is a transcriptional repressor of these genes. Furthermore, since the small RNA Yfr1 is involved in cell-wall remodeling through the modulation of several porins, including *amiC2* and Omp85 (42), the potential binding of FurC to *yfr1* promoter region was tested, yielding a positive result.

**TABLE 1:** Cell-wall-related proteins with lower abundance in the exoproteome of FurC-overexpressing strain with respect to that of *Anabaena* sp. strain PCC 7120

ORF	Protein name; description	Log_2_FC	EMSA^*a*^
*alr4067*	LptA; lipopolysaccharide export system protein	−4.11	+
*alr2887*	HgdD; heterocyst glycolipid deposition protein	−3.90	+
*alr3345*	Hypothetical protein; CsgG family	−3.83	
*alr0093*	AmiC2; N-acetylmuramoyl-L-alanine amidase	−3.69	+
*alr3276*	Probable outer membrane peptidase	−3.58	
*alr1819*	Hypothetical protein, fasciclin and S-layer homology domains	−3.50	
*alr3608*	Abp3; putative S-layer associated multidomain endoglucanase	−3.33	
*all0495*	Polysaccharide biosynthesis/export protein	−3.32	
*alr0092*	AmiC1; N-acetylmuramoyl-L-alanine amidase;	−3.23	+
*all4499*	Probable carbohydrate-selective porin; OprB family	−3.13	+
*alr2269*	Omp85; outer membrane protein insertion porin family	−3.12	+
*alr4550*	Probable carbohydrate-selective porin; OprB family	−3.00	−
*alr4893*	Outer membrane protein insertion porin family	−3.00	
*all7614*	Probable carbohydrate-selective porin; OprB family	−2.97	
*alr0834*	OprB-I; major outer membrane protein	−2.86	−
*all4999*	N-acetylmuramoyl-L-alanine amidase	−2.84	+
*alr4741*	Probable carbohydrate-selective porin; OprB family	−2.73	−

^*a*^ Genes whose promoter region was tested by EMSA with FurC showing the result as ±.

### Overexpression of *furC* in *Anabaena* dramatically impairs septal nanopore formation and intercellular molecular transfer

**Fig 6:**
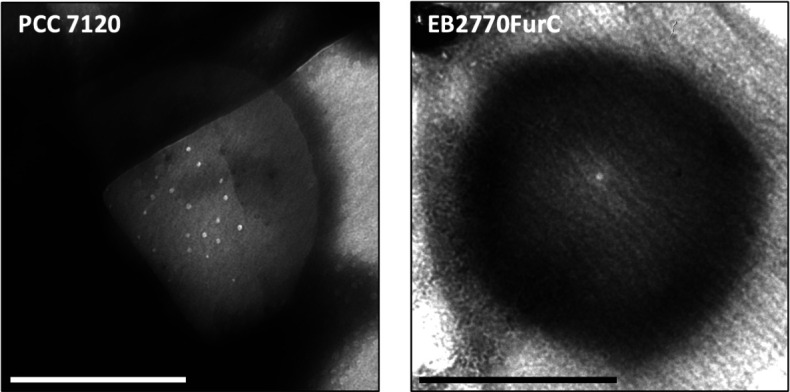
Nanopore array example of septal PG disks of WT *Anabaena* sp. strain PCC 7120 and the *furC*-overexpressing strain EB2770FurC. Cells grown until the exponential phase were used for the isolation of PG and visualization by transmission electron microscopy, as described in Materials and Methods. Photographs are representative of between 10 and 13 PG disks photographed for each strain. Scale bars, 0.5 µm.

The amidases AmiC1 and AmiC2 are responsible for the formation of the nanopores in the septal PG disks, which facilitate intercellular molecular transfer. Deletion strains of these genes display fewer nanopores and decreased intercellular communication (35). Since FurC regulates *amiC1*, *amiC2*, and several cell-wall-related genes (Fig. 4) whose protein products decreased in the exoproteome fraction of the *furC*-overexpressing strain (Table S2), murein sacculi (PG) of WT and EB2770FurC strains were isolated and examined. As observed in Fig. 6; Fig. S3, the WT *Anabaena* displayed an average of 21 ± 7 (*n* = 10) nanopores per septal disk, consistent with the typically reported number for this strain under similar conditions (i.e., BG11 medium) (35, 43–45). However, the septal PG disks of EB2770FurC exhibited a distinct phenotype, with a single central nanopore being the most common alteration (7/13), and even PG disks showing no nanopore at all (2/13; Fig. 6; Fig. S4). Out of the additional four PG disks analyzed, three had a single large “nanopore,” which has been associated with a septum in the process of formation (46, 47), and one presented aberrant nanopores (Fig. S4).

**Fig 7:**
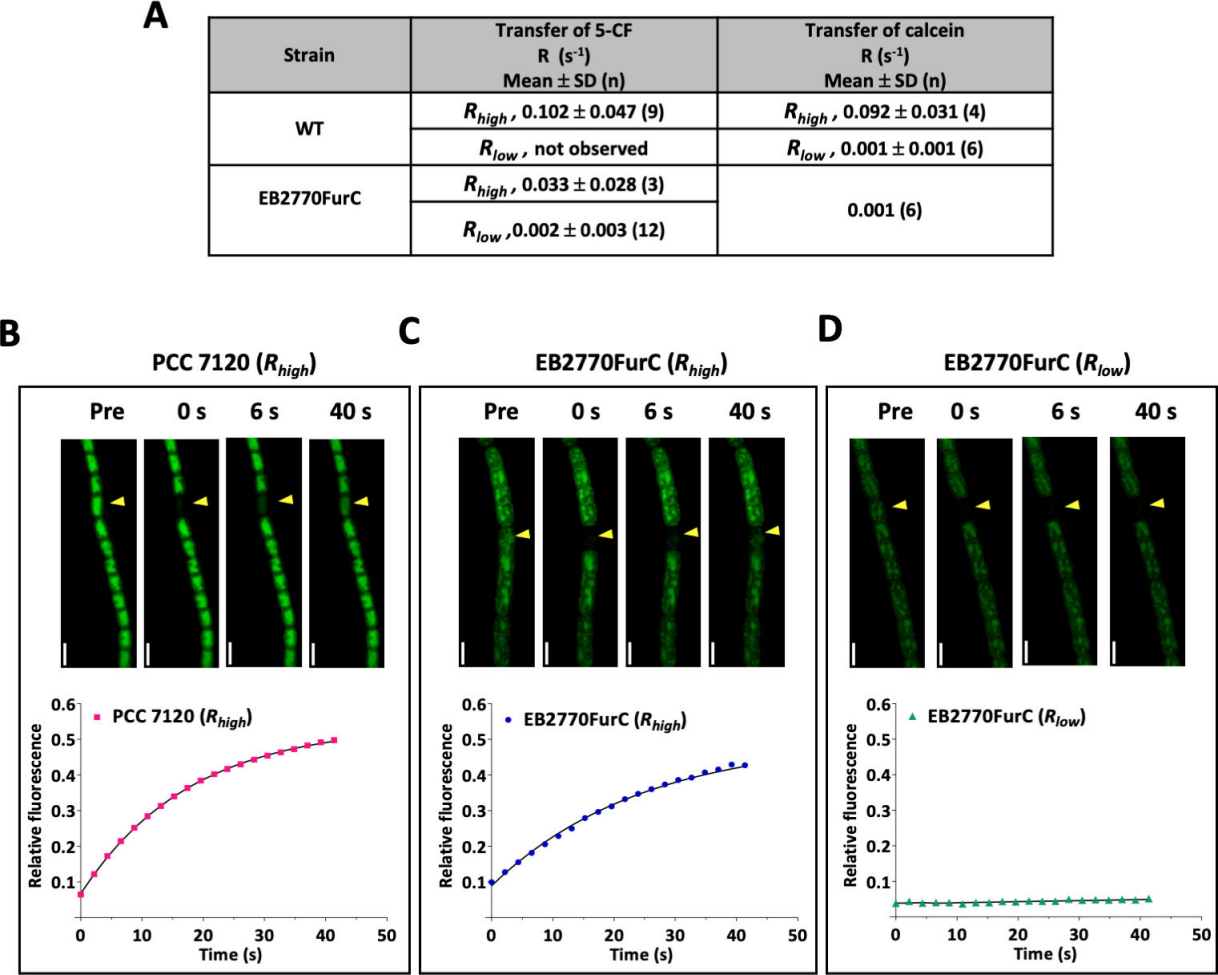
FRAP analyses of *Anabaena* sp. strain PCC 7120 and EB2770FurC. (**A**) Rate constants (***R***) of 5-CF and calcein recovery from FRAP analysis in *Anabaena* sp. PCC 7120 (WT) and the *furC-*overexpressing strain (EB2770FurC). Note the existence of two populations of cells, communicating cells (*R*_high_ = *R* ≥ 0.01 s^−1^) and noncommunicating cells (*R*_low_ = *R* ≤ 0.01 s^−1^) observed for 5-CF and calcein transfer in the EB2770FurC and the WT, as previously described and discussed in the study by González et al. (38). 5-CF transfer in the WT showed all communicating cells. Filaments were labeled with 5-CF and calcein and subjected to FRAP analysis as described in Materials and Methods. (**B, C, and D**) Examples of 5-CF FRAP experiments of (**B**) *Anabaena* sp. PCC 7120 WT and (**C and D**) EB2770FurC. The cell indicated by arrowheads was photobleached, and its fluorescence was monitored continuously. Images prior (pre) and at 0, 6, and 40 s after bleaching are shown in the upper panel. Scale bars, 4 µm. Fluorescence recovery curves for their respective bleached cells are shown below.

The observed nanopore structures in EB2770FurC prompted us to analyze potential alterations in the intercellular communication between the cells within individual filaments of this strain. This was tested by measuring the intercellular transfer of 5-carboxyfluorescein (5-CF) and calcein between vegetative cells of BG11-grown cultures by fluorescence recovery after photobleaching (FRAP) analysis. Fig. 7 shows that, in contrast to the values usually obtained for WT *Anabaena* (44), the *furC*-overexpressing strain showed an exceptionally high percentage of noncommunicating cells. In fact, the recovery rate of fluorophore was completely abolished (*R* < 0.01 s^−1^) in 80% of cells for 5-CF and 100% for calcein. Further analysis showed that though EB2770FurC filaments displayed 20% of communicating cells for 5-CF, the recovery rate decreased to 30% when compared to values reached with *Anabaena* filaments, indicating that EB2770FurC not only showed a higher percentage of noncommunicating cells but also that the intercellular transfer between the communicating cells was affected in this strain.

## DISCUSSION

The composition of the extracellular milieu defines microbial interactions with the environment-modulating processes such as intercellular communication, toxin production, or biofilm formation, tailoring bacterial ability to cope with different stresses (28, 48, 49). Previous studies point to FurC as an essential stress regulator, playing a central function in the modulation of peroxide, as well as light and nitrogen responses in *Anabaena* (30, 31, 50–52). Furthermore, genome-wide identification of novel direct FurC targets unveiled the role of this protein in the regulation of central carbon metabolism (53). The FurC regulon also includes several genes encoding transporter components that are important contributors to cell molecular exchange with the environment, such as *alr4028-33* (Fec system) operon, *hgdC and zupT* (30, 53). Consequently, the deregulation of FurC targets causes important phenotype alterations in *furC*-overexpressing cells, which include destabilization of the photosynthetic machinery, altered cell morphology and division, and failure in heterocyst development under nitrogen-fixing conditions (30, 31, 53).

Considering the central role of FurC in *Anabaena*, we sought to investigate the effect of FurC deregulation on the composition of the exoproteome and the exometabolome of this cyanobacterium. SWATH-MS proteomic analysis allowed us to identify 96 proteins with significant changes in abundance, being 67 of them less abundant in the exoproteome of *furC*-overexpressing cells. This fact, together with the absence of bluish pigments along isolation of the extracellular fractions, was indicative of cell integrity.

Thus, the SWATH results support the claim that the overexpression of FurC directly or indirectly affected the abundance of proteins in the exoproteome of *Anabaena* involved in various functional processes, including those in which FurC executes direct regulation at the transcriptional level, such as the oxidative stress response or photosynthesis and respiration. However, it is important to note that the abundance of these proteins in the exoproteome not only depends on their transcription but may also involve posttranscriptional regulation, as well as secretion and accumulation processes in the cellular medium. Actually, the correlation between the transcriptome and proteome is quite poor (54), indicating that the posttranscriptional processes are key players in adapting to new environments. It is not surprising then that of the 96 proteins whose relative abundances were different in both strains, only 12 of them showed differences in gene expression in the *furC*-overexpressing strain with respect to the WT *Anabaena*, according to transcriptional analysis previously performed (Table S2) (30, 31). Furthermore, within these, only six genes showed the same trend of increase or decrease in both their abundance and gene expression.

The higher abundance in the EB2770FurC exoproteome of proteins involved in relieving oxidative stress, such as PrxA, flavodoxin (IsiB), or the Dps homolog All1173, could be a compensatory strategy to overcome the higher stress experienced by *furC*-overexpressing cells (31). Conversely, the amount of FurA was substantially lower in the exoproteome of *furC*-overexpressing cells compared to the wild-type strain. The FurA ortholog has been detected previously in the peripheral membrane-associated fractions of *Synechocystis* sp. PCC 6803 and *Anabaena* cells (55). Additionally, it was demonstrated that the levels of FurA (Fur) in *Synechocystis* are regulated in response to iron deficiency through direct proteolysis by the essential membrane-embedded FtsH1/3 complex (55, 56). Nevertheless, in these studies, the levels of this regulator in the extracellular fraction were not inspected (55, 56)

Similarly, a decrease in the abundance of other proteins related to the cell wall was observed. Among them is the membrane transporter TolC homolog, HgdD, which has been described as the major component of the metabolite export system in *Anabaena* (39, 57–59). In fact, as a modulator of the protein secretion pattern, it was observed that an *hgdD* deletion mutant showed significant differences in the secretome of *Anabaena* and higher activity of enzymes involved in ROS detoxification (39, 60). Therefore, it cannot be ruled out that some of the differences observed in the exoproteome of the *furC*-overexpression strain are due to the decrease in HgdD levels. Other cell-wall-related proteins that were less abundant in the exoproteome of the *furC-*overexpressing strain included outer membrane proteins OprB1 (Alr0834), Omp85 (Alr2269), the OprB-like proteins Alr4499 and Alr4550, LptA (Alr4067), and the murein amidases AmiC1 (Alr0092) and AmiC2 (Alr0093). Most of these genes are modulated by the small RNA Yfr1, thus contributing to the control of cell-wall homeostasis (42), as well as by FurC (Fig. 4). The fact that FurC recognizes and binds to *yfr1* promoter region discloses a new potential indirect modulation by this regulator of Yfr1 targets.

Furthermore, AmiC1 and AmiC2 are involved in the remodeling of the PG layer and the formation of nanopores. The exchange of metabolites between *Anabaena* cells in the filament takes place through the SJs that connect the cytoplasm of adjacent cells, which traverse the PG through perforations called nanopores (61, 62). Our results show that *amiC1* and *amiC2* are directly downregulated by FurC, and the lower levels of these proteins in the exoproteome of EB2770FurC are correlated with the impairment of septal nanopore formation and intercellular molecular transfer in EB2770FurC cells. Similarly, *amiC1* and *amiC2* deletion mutants showed a significant reduction in the number of nanopores that affected intercellular communication, cellular morphology, and heterocyst differentiation (35). Hence, alterations in the phenotype of EB2770FurC cells, such as the failure to form heterocysts or alterations in cellular morphology, could be clearly linked to the downregulation of *amiC* genes (30, 31).

Likewise, the observed involvement of FurC in the regulation of intercellular transfer would be consistent with the blockage of intercellular communication that has been reported during stress induction, particularly under oxidative stress. While the modulation of gating of septal junctions was proposed as a mechanism for fast control of intercellular molecular transfer, an additional route involving the regulation of expression of nanopore formation factors, as observed in this study, could be also considered. Given the similar scenario of the *furC*-overexpressing strain with the *furC* induction in the wild-type strain in response to oxidative stress induced by H_2_O_2_ or methyl viologen (51, 52), it is plausible to hypothesize that under such conditions, FurC may be playing a key role in the regulation of the molecular transfer between cells in response to stress.

It should be taken into account that the expression of other FurC targets related to the stress response, as well as genes that are not direct FurC targets, will be affected in the *furC*-overexpressing variant, resulting in stressed cells and disturbed cell homeostasis, including cell envelope homeostasis. Although the integrity of the outer membrane seems to be compromised in EB2770FurC cells, the potential leakage of periplasmic components due to FurC overexpression is under the level of detection using SWATH-MS since no periplasmic proteins have been identified as more prevalent in the exoproteome of *furC*-overexpressing cells compared to the wild-type *Anabaena*.

Another interesting feature of the EB2770FurC strain is the amount of peptidoglycan fragments released into the media (Fig. 3). Similar scenarios have been reported in two mutants of *Bacillus subtillis* carrying deletions of genes encoding NamZ or NagZ (63, 64), though no orthologs of either NamZ or NagZ are found in *Anabaena*. While the Δ*nagZ* was reported to accumulate both GlcNAc-anhMurNAc and GlcNAc-MurNAc in the culture media, none of the mutants reported to date specifically release the two anhydrosugar fragments (63). Another possibility is that the abundance of extracellular anhydro-based muropeptides in EB2770FurC plays a signaling role. Recent studies point to PG muropeptides as signaling molecules since multiple structural motifs and proteins have been described to bind PG (40). Besides, other potential roles, including involvement in microbial interactions, symbiotic associations, and pathogenesis in animals and plants, have been proposed (40).

In summary, our findings demonstrate that *furC* overexpression shapes the extracellular composition of *Anabaena* cells, affecting both the exoproteome and the exometabolome and unveiling the involvement of FurC in the modulation of cell-to-cell transfer, while also suggesting a potential role in the regulation of broader processes of cell-wall biogenesis and PG recycling.

## MATERIALS AND METHODS

### Strains and growth conditions

*Anabaena* sp. (also known as *Nostoc* sp.) strain PCC 7120 and its genetically modified strain EB2770FurC (31) were grown photoautotrophically in BG11 medium (65) or BG11C medium (BG11 supplemented with 8.8 mM of NaHCO_3_) at 28°C under constant illumination of 30 μE m^−2^ s^−1^ in an orbital shaker at 130 rpm. Cultures of EB2770FurC contained 50 µg·mL^−1^ of neomycin. To test the membrane integrity of EB2770FurC cells, liquid cultures of EB2770FurC and the controls wild type and an Nm-resistant strain that harbors a pRL25C-derived plasmid expressing the mVenus protein (López-Igual and Luque, unpublished) were grown in BG11C media and set up at OD_750_ = 1.0, and 5 µL of serial twofold dilutions was spotted on BG11C plates or BG11C plates supplemented with 25 µg·mL^−1^ of Nm containing the different harmful compounds as indicated in Fig. 4; Fig. S4. Plates were incubated under constant illumination (30 µmol photons m^−2^ s^−1^) at 30°C for 6 days.

### Culture setup for exoproteome and exometabolome isolation

Cultures of two biological replicates of WT *Anabaena* and the *furC*-overexpressing strain were started from plates in 40 mL of BG11C medium. Cells were harvested by gentle centrifugation at room temperature at the late exponential phase of growth. These cells were used to set up 80 mL cultures of each replicate at an OD_750_ of 0.15 in BG11C medium. Cultures were grown until they reached OD_750_ = 1–1.5 (about 12 days) and sequentially filtered through 0.45 and 0.22 µm with mixed cellulose esters (MCE) filters (Filter-Lab). The final flow-through was divided for two purposes: 10 mL for metabolomic analyses and between 50 and 60 mL for the proteomic analyses. Samples were immediately frozen at −80°C and lyophilized. For exoproteome analyses, the lyophilized material was resuspended in 2.5 mL of buffer A (50 mM Tris-HCl pH 7.5 and Complete EDTA-free protease inhibitor cocktail) and dialyzed against 3 L of buffer A, performing two overnight steps to get rid of precipitated salts from BG11 medium that prevented the correct analysis. After dialysis, the sample was transferred to 15 mL tubes, frozen at −80°C, and lyophilized. A series of precautions were applied during all stages of culturing sample manipulation to reduce the levels of contaminants (surfactants, polysiloxanes, and polyethylene glycols) that could affect the metabolomic work. The Erlenmeyer flasks were rinsed thoroughly with Milli-Q water and sterilized in autoclave bags. All plastic material, except tips, was rinsed twice with 40% methanol in Milli-Q water, dried in an oven at 50°C, and sterilized by exposure to UV light for 30 min in a laminar flow hood. All the filters were rinsed thoroughly by filtering 30–40 mL of 40% methanol in Milli Q water and allowed to dry in an oven at 50°C.

### Exoproteome analysis

Mass spectrometry analyses were carried out at the Proteomics Service of the Instituto de Bioquímica Vegetal y Fotosíntesis (Seville, Spain). The analyses were performed in a triple quadrupole time of flight (TOF) hybrid mass spectrometer (5600 plus, Sciex), equipped with a nanospray source coupled to an Eksigent model 425 nanoHPLC. The mass spectrometry proteomics data have been deposited to the ProteomeXchange Consortium (http://proteomecentral.proteomexchange.org) via the PRIDE partner repository (66) with the data set identifier PXD043659. Control of the equipment and acquisition and processing of data were performed using Analyst TF 1.7 software. The methodology for sample processing and the following pipeline for the creation of spectral ion libraries, SWATH-MS quantification, and final data processing and statistical analyses are compiled in the supplemental methods in File S1.

Functional categorization of DAPs found in the exoproteome was performed according to the COG classification provided by EGGNOG mapper (67). The name and predicted function were checked manually in KEGG and Uniprot (68) databases to correct biases. Prediction of signal peptides was performed with SignalP-6.0 software (69) available at https://services.healthtech.dtu.dk/services/SignalP-6.0/. Prediction of secretion was performed with SecretomeP 2.0 (70) and BastionX from Bastion Hub suite (71) available at https://services.healthtech.dtu.dk/services/SecretomeP-2.0/ and https://bastionhub.erc.monash.edu/bastionxPrediction.jsp. Prediction of subcellular localization was performed with the PSORTb software version 3.0.3 (72) available at https://www.psort.org/psortb/.

### Exometabolome analyses

The lyophilized samples were resuspended in 9:1:0.001 (water/acetonitrile/formic acid; vol/vol/vol), with volumes normalized to conditioned media (approximately 9.4 × 10^8^ cells/mL). The samples were vortexed briefly, sonicated for 10 min, and centrifuged (13,000 × *g*, 10 min). Aliquots (200 µL) were then transferred to LC–MS vials for analyses, which were performed on a Shimadzu LCMS9030 high-resolution QToF coupled to a Nexera LC40 UHPLC system. Samples (2 µL) were injected into a reversed-phase column (Waters BEH C_18_, 1.7 μm, 2.1 × 50 cm) and subjected to a gradient separation at 400 µL/min. Solvent A was water containing 0.1% formic acid, and solvent B was methanol containing 0.1% formic acid. The initial mobile phase was 95:5 (A:B), and at 1.5 min, a linear gradient was invoked, which ended at 95% solvent B at 10 min. This was held for 2 min and ramped back down to 5% solvent B over a period of 1 min and subsequently held for 2 min at 5% B for reequilibration. The mass spectrometer acquired data from 0.75 to 15 min using positive ion profile mode from 150 to 2,000 *m/z* using electrospray ionization (4.0 kV) and a scan time of 0.1 s. Gas flows were 2, 10, and 10 L/min (nebulizing, heating, and drying, respectively), with interface (300°C) and desolvation (526°C) and DL (250°C) temperatures as indicated.

### Real-time RT-PCR assays

Total RNA used for real-time RT-PCR was obtained from three biological replicates of *Anabaena* WT and EB2770FurC cultured for 12 days, following the same steps and conditions as for the exoproteome and exometabolome cultures stated above. RNA extraction and real-time RT-PCR analyses were carried out as described in the study by Sarasa-Buisan et al. (30). The sequences of specific primers designed with Primer Express 3 (ThermoFisher) are shown in Table S1. Transcript levels of target genes were normalized to those of the housekeeping gene *rnpB* measured with the same samples (73). Relative quantification and expression fold changes were calculated according to the comparative Ct method (ΔΔCt method) (74). The fold change threshold was set up to ±1.5-fold.

### Electrophoretic mobility shift assays

DNA fragments for EMSA were obtained by PCR, using the *Anabaena* genome as a template and the primers listed in Table S1 to amplify 250–350 bp of the promoter regions of selected genes. EMSA analyses with FurC were performed as previously described (30) including 100 µM MnCl_2_ in both gel and running buffer. Gels were stained with SYBR Safe (Invitrogen) and visualized in a GelDoc 2000 device (Bio-Rad). In all assays, the specific binding to the previously recognized FurC target, *hetZ* (30), was used as a positive control.

### Nanopore analysis

Filaments grown to OD_750_ = 0.6–0.8 were collected by gentle centrifugation at room temperature, and the PG sacculi were isolated and analyzed as previously described (45). The purified PG sacculi were placed on formvar/carbon film-coated copper grids and stained with 1% (wt/vol) uranyl acetate. Samples were visualized with a Zeiss Libra 120 Plus electron microscope at 120 kV (Servicio de Microscopía, Universidad de Sevilla, Seville, Spain).

### FRAP experiments

Calcein and 5-CF labeling were performed as reported previously (75, 76). All measurements were carried out at 30°C. Images were collected with an Olympus FLUOVIEW FV3000 confocal laser-scanning microscope equipped with a UPlanApo 60× 1.5 NA oil immersion objective. Fluorescence emission was monitored by collection across windows of 500–520 nm and a 150 µm pinhole. After an initial image was recorded, the bleach was carried out by a preset FRAP routine previously described (75). Postbleach images were taken in XY-mode approximately every 2 s over a time of 40 s. Kinetics of calcein and 5-CF transfer were computed with Fiji processing package from ImageJ (77), and the recovery constant, R, was calculated as previously described (78) for both fluorescent tracers.

## Data Availability

The mass spectrometry proteomics data have been deposited to the ProteomeXchange Consortium (http://proteomecentral.proteomexchange.org) via the PRIDE partner repository (66) with the data set identifier PXD043659.

## SUPPLEMENTAL MATERIAL

Figure S1 mbio.03231-23-s0001.pdf MS/MS fragmentation data for anhydroMurNAc and GlcNAc-anhydroMurNAc.

Figure S2 mbio.03231-23-s0002.pdf Assessment of the outer membrane integrity in EB2770FurC cells vs the NmR-control strain.

Figure S3 mbio.03231-23-s0003.pdf Total images of nanopores in septal peptidoglycan disks of *Anabaena* WT.

Figure S4 mbio.03231-23-s0004.pdf Total images of nanopore arrays in septal peptidoglycan disks of EB2770FurC

Additional experimental details mbio.03231-23-s0005.docx Supplemental methods for exoproteome analysis.

Table S1 mbio.03231-23-s0006.docx Oligonucleotides used in this study.

Table S2 mbio.03231-23-s0007.xlsx Differentially abundant proteins detected by SWATH-MS in the exoproteome fraction of EB2770FurC compared to that of *Anabaena* sp. PCC7120.

ASM does not own the copyrights to Supplemental Material that may be linked to, or accessed through, an article. The authors have granted ASM a non-exclusive, world-wide license to publish the Supplemental Material files. Please contact the corresponding author directly for reuse.
