# Supplementary figures and images for: FurC (PerR) contributes to the regulation of peptidoglycan remodeling and intercellular molecular transfer in the cyanobacterium Anabaena sp. strain PCC 7120
Source: mBio. 2024 Feb 9;15(3):e03231-23. doi: 10.1128/mbio.03231-23 (PMC10936207; doi:10.1128/mbio.03231-23)

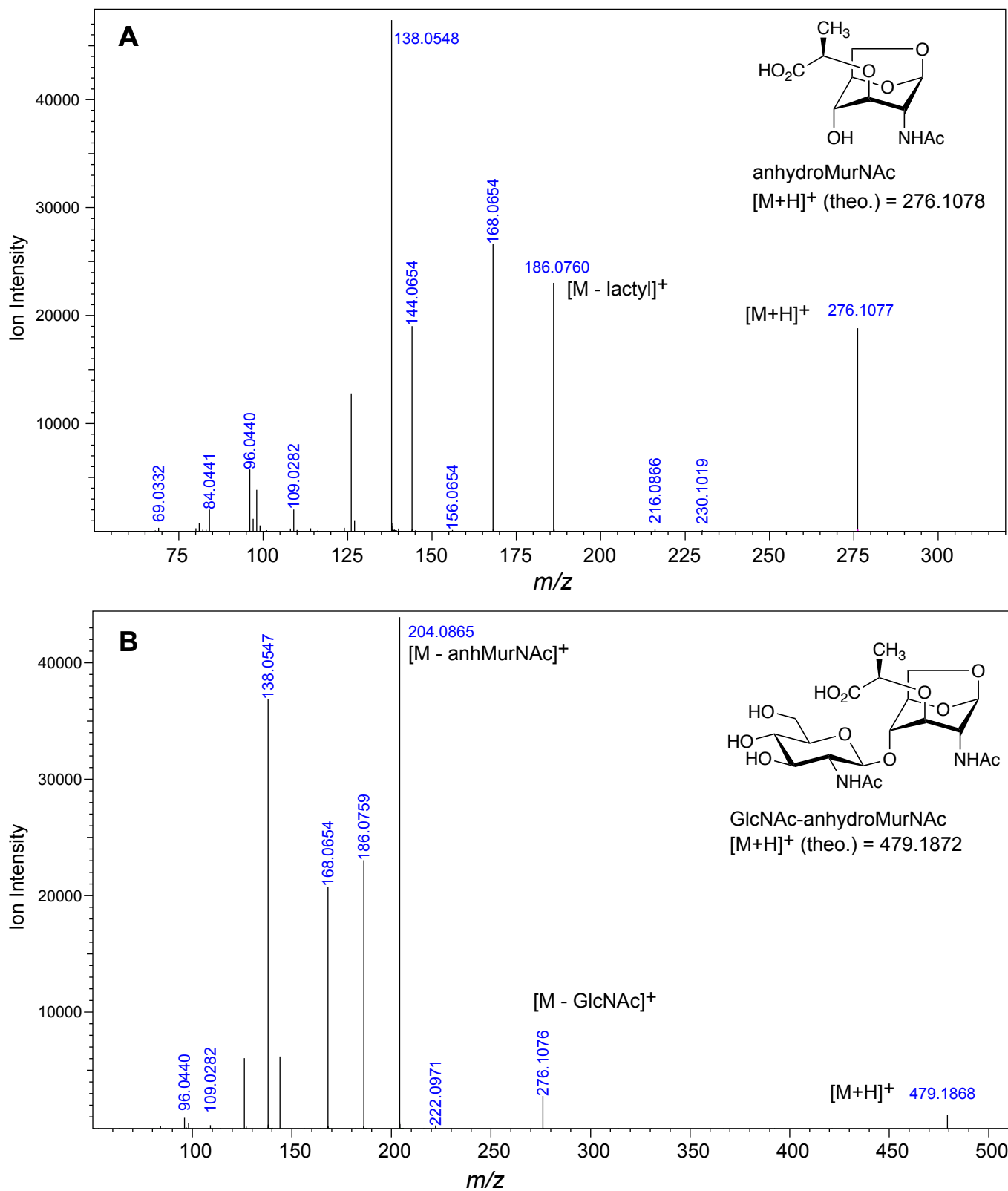

**Figure S1.** MS/MS fragmentation data for anhydroMurNAc (**A**) and GlcNAc-anhydroMurNAc (**B**).

Supplement: Figure S1 — MS/MS fragmentation data for anhydroMurNAc and GlcNAc-anhydroMurNAc. [file mbio.03231-23-s0001.pdf]
