# Supplementary material for: FurC (PerR) contributes to the regulation of peptidoglycan remodeling and intercellular molecular transfer in the cyanobacterium Anabaena sp. strain PCC 7120
Source: mBio. 2024 Feb 9;15(3):e03231-23. doi: 10.1128/mbio.03231-23 (PMC10936207; doi:10.1128/mbio.03231-23)
Supplement: Figure S2 — Assessment of the outer membrane integrity in EB2770FurC cells vs the NmR-control strain. [file mbio.03231-23-s0002.pdf]

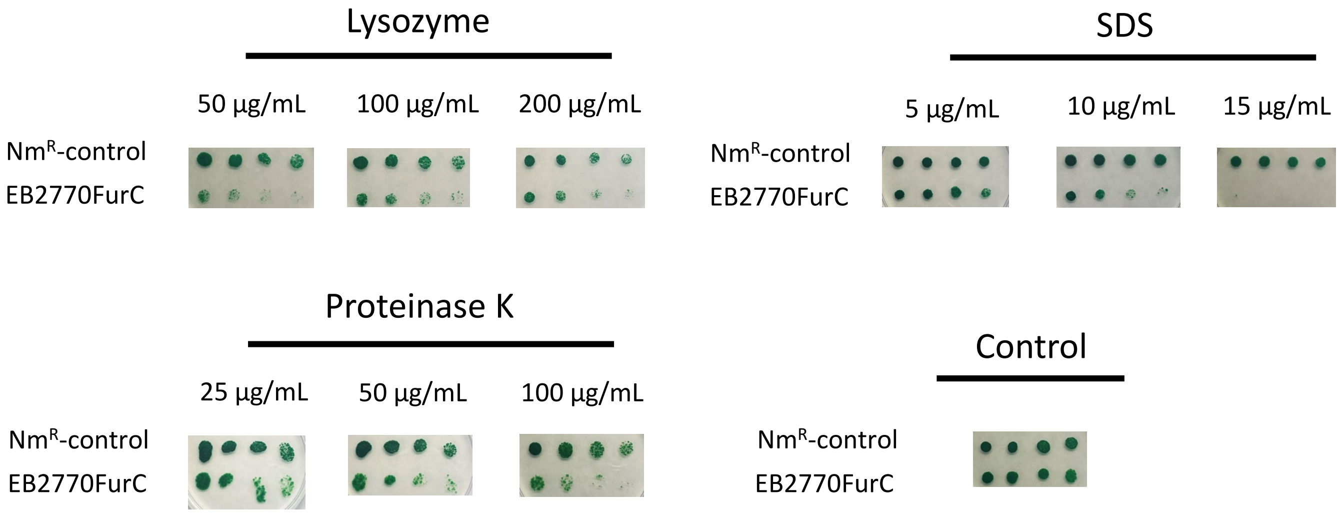

Figure S2. Assessment of the outer membrane integrity in EB2770FurC cells versus the Nm^R-control strain. Representative images of the outer membrane integrity assays using 5 µl of serial 2-fold dilutions of Nm^R-control strain and the *furC*-overexpressing strain EB2770FurC at OD_{750} = 1.0 were spotted onto media composed of BG11C supplemented with 25 µg·mL^{-1} of neomycin and the indicated substances. Images were taken after 6 days of growth.
