# Supplementary material for: FurC (PerR) contributes to the regulation of peptidoglycan remodeling and intercellular molecular transfer in the cyanobacterium Anabaena sp. strain PCC 7120
Source: mBio. 2024 Feb 9;15(3):e03231-23. doi: 10.1128/mbio.03231-23 (PMC10936207; doi:10.1128/mbio.03231-23)
Supplement: Figure S3 — Total images of nanopores in septal peptidoglycan disks of Anabaena WT. [file mbio.03231-23-s0003.pdf]

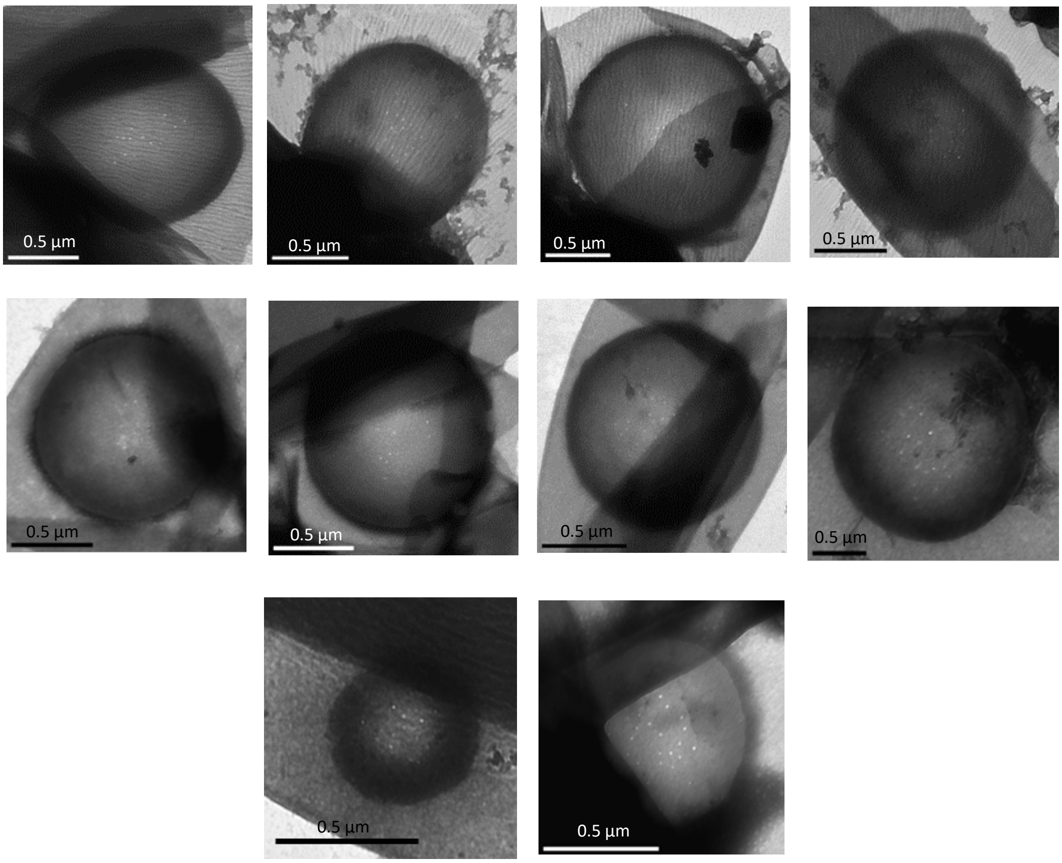

Supplementary Figure S3. Total images of nanopores in septal peptidoglycan disks of *Anabaena* WT. The PG was isolated and visualized by transmission electron microscopy as described in Materials and Methods. The calculated mean of nanopores per septal PG disk is 21 ± 7 (n=10).
