# Supplementary material for: FurC (PerR) contributes to the regulation of peptidoglycan remodeling and intercellular molecular transfer in the cyanobacterium Anabaena sp. strain PCC 7120
Source: mBio. 2024 Feb 9;15(3):e03231-23. doi: 10.1128/mbio.03231-23 (PMC10936207; doi:10.1128/mbio.03231-23)
Supplement: Figure S4 — Total images of nanopore arrays in septal peptidoglycan disks of EB2770FurC [file mbio.03231-23-s0004.pdf]

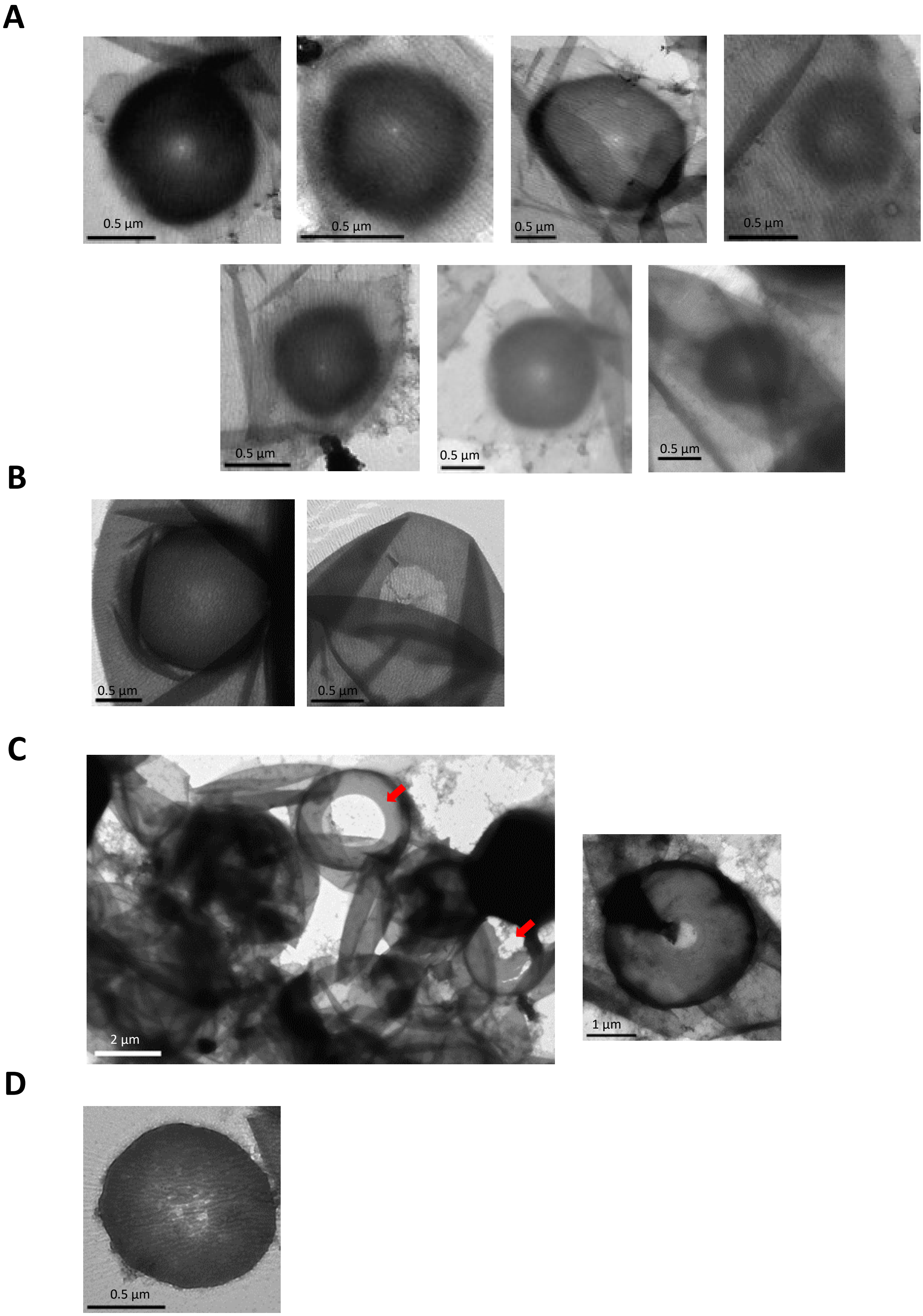

Supplementary Figure S4. Total images of nanopore arrays in septal peptidoglycan disks of EB2770FurC. Images depict several phenotypes: A) One nanopore per septal disk, B) Absence of nanopores, C) Single large "nanopore", which has been associated with a septum in process of formation C) Aberrant array of nanorpores. The PG was isolated and visualized by transmission electron microscopy as described in Materials and Methods.
