# Supplementary material for: FurC (PerR) contributes to the regulation of peptidoglycan remodeling and intercellular molecular transfer in the cyanobacterium Anabaena sp. strain PCC 7120
Source: mBio. 2024 Feb 9;15(3):e03231-23. doi: 10.1128/mbio.03231-23 (PMC10936207; doi:10.1128/mbio.03231-23)
Supplement: Additional experimental details — Supplemental methods for exoproteome analysis. [file mbio.03231-23-s0005.docx]

**Supplementary File S1.** Supplementary methods for exoproteome analysis

*Sample processing and mass spectrometry analyses*

Lyophilized samples were resuspended in 800 µL of buffer containing 50 mM Tris-HCl pH 7.5; 1 mM PMSF, 1 mM EDTA, 2 % SDS and a cocktail of protease inhibitors and were centrifuged at 20000 g for 2 min to eliminate particles. The supernatants were subjected to precipitation with trichloroacetic acid (TCA)/acetone. Subsequently, sample digestion was performed as follows; the precipitated extracts were resuspended in 20 µL of 50 mM ammonium bicarbonate with 0.2 % Rapidgest (Waters) and the amount of proteins was quantified using Qubit system. 10 µg of total protein was incubated with 4.5 mM DTT for 30 min at 60 ˚C and then with 10 mM chloroacetamide, 30 min in darkness at room temperature. Trypsin treatment was performed at 37 ˚C O/N in a ratio 1:40 (Trypsin:Protein). Digestion was stopped by addition of formic acid. A mixture of SCIEX synthetic peptides was also added to each sample to a final concentration of 50 femtomole/µL, for posterior normalization of chromatograms necessary for SWATH processing. A volume equivalent to 1 µg of protein was injected in LC-MS/MS Peptides were first loaded onto a trap column (Acclaim PepMap 100 C18, 5 µm, 100 Å, 100 µm id × 20 mm, Thermo Fisher Scientific) isocratically in 0.1 % formic acid/2 % acetonitrile (v/v) at a flow rate of 3 μL/min for 10 min. Subsequently, they were eluted on a reverse phase analytical column, Acclaim PepMap 100 C18, 3 µm, 100 Å, 75 µm id × 150 mm, Thermo Fisher Scientific, coupled to a PicoTip emitter (F360-20-10-N- 20_C12 from New Objective). Peptides were eluted with a linear gradient of 2-35 % (v/v) of solvent B in 60 min at a flow rate of 300 nL/min. As solvents A and B, formic acid 0.1% (v/v) and acetonitrile with formic acid 0.1 % (v/v) were used, respectively. The voltage source was set at 2600 V and the heater temperature remained at 100 ˚C. Gas 1 was selected at 15 psi, gas 2 to zero, and the curtain to 25 psi. For the protein identification necessary for the creation of spectral ion libraries for SWATH-MS quantification, the acquisition was carried out with a DDA method (data dependent acquisition), consisting of a TOF-MS Scan between 400-1250 m/z, accumulation time of 250 ms, followed by 50 MS/MS (230 -1500 m/z), accumulation time of 65 ms and with a total 3,54 s cycle time. Protein identification was performed using the ProteinPilot™ software (version 5.0.1, Sciex) with the Paragon™ Algorithm. The search was conducted against the UniProt proteome *Nostoc* sp. PCC7120 FASTA (15/07/2021) combined with the SCIEX contaminants database. Automatically generated reports in ProteinPilot were manually inspected for false discovery rate (FDR); cut-off proteins with only proteins identified at an FDR ≤1% were considered for subsequent analyses. SWATH experiments were performed using three technical replicates of each biological replicate (1 µg protein) using a DIA method (data-independent acquisition). The method consisted of repeated acquisition cycles of time-of-flight mass spectrometry TOF MS/MS scans (230–1,500 m/z, 60 ms acquisition time) of 60 overlapping sequential precursor isolation windows of variable width (1 m/z overlap) covering the 400–1,250 m/z mass range from a previous TOF MS scan (400–1,250 m/z, 50 ms acquisition time) for each cycle with 3.68 s total cycle time. Autocalibration of the equipment and chromatographic conditions were controlled by an injection of a standard Pepcalmix (Sciex), between the replicates.

*SWATH data processing and statistical analysis*

SWATH-MS spectral alignment was performed using the PeakView 2.2 software (Sciex) with the MicroApp SWATH 2.0 using the spectral library generated through DDA method (1% FDR). Normalization of protein intensity was performed with MarkerView software (version 1.2.1.1, AB Sciex). The R package limma v3.54.1 (67) was used to assess the significant differences in abundance of exoproteins between EB2770FurC and *Anabaena* sp. PCC7120. Briefly, normalized data were log2-transformed, median centered and the three technical replicates averaged before fitting the data to a linear model (lmFit, robust method), and using moderated t-statistics with Limma's empirical Bayes method to analyze differential expression. The p-values were adjusted for multiple hypotheses testing using Benjamini-Hochberg FDR correction. Proteins were classified as differentially abundant proteins (DAPs) if the adjusted p-value (q-value) was less than 0.001.
