# Supplementary material for: FurC (PerR) contributes to the regulation of peptidoglycan remodeling and intercellular molecular transfer in the cyanobacterium Anabaena sp. strain PCC 7120
Source: mBio. 2024 Feb 9;15(3):e03231-23. doi: 10.1128/mbio.03231-23 (PMC10936207; doi:10.1128/mbio.03231-23)
Supplement: Table S1 — Oligonucleotides used in this study. [file mbio.03231-23-s0006.docx]

| **Supplementary Table S1.** Oligonucleotides used in this study | | |
| --- | --- | --- |
| Primer | Sequence (5´- 3´) | Purpose |
| *EMSA* | | |
| Palr4067_up | CGGTAACAGATAATTAGTTAATC | Promoter region of *alr4067* (*lptA*) |
| Palr4067_dw | CAGAGGCAATTTATAGCAG |  |
| Palr2887_up | ATTGTCAGTGGCTCTCCTA | Promoter region of *alr2887* (*hgdD*) |
| Palr2887_dw | GGCAAGAAACTATAGAATAAG |  |
| Palr0093_up | CCTCAAACGATAGATAATCAG | Promoter region of *alr0093* (*amiC2*) |
| Palr0093_dw | CACAATCTTTTCTCCTGATG |  |
| palr0092_up | GTCATAAAATAACTCAATTC | Promoter region of *alr0092* (*amiC1*) |
| palr0092_dw | CAATCCATTCTCCTGATTTG |  |
| Pall4499_up | GTCGTATTATTCTATGTTTAC | Promoter region of *all4499* |
| Pall4499_dw | GTTCCAGAATAGTTTTGTC |  |
| Palr2269_up | GACCTGGGAAATGTAGAAG | Promoter region of *alr2269* (*omp85*) |
| Palr2269_dw | CTCAAGGGTGCTGTGATTG |  |
| Palr4550_up | GGAGTTGTAAGTTAAATACTC | Promoter region of *alr4550* |
| Palr4550_dw | CATTTTTACTTTTCTCCTCAC |  |
| Palr0834_up | GCTATTAAGGACATGATGCCATTTTG | Promoter region of *alr0834* (*oprB-I*) |
| Palr0834_dw | CTTAGCAATTTTGTCACATTAAAAGTC |  |
| Pall4999_up | GAACCAGTGGTAACACTATTG | Promoter region of *all4999* |
| Pall4999_dw | GACTGTGGCACAATGACGG |  |
| Palr4741_up | GTAGCCCGCGATCGCTAATC | Promoter region of *alr4741* |
| Palr4741_dw | ATCACTCCTCACACCTGATC |  |
| FurCbox_yfr1_up | CTAGATGATGGTACTCATAAC | Promoter region of sRNA yfr1 |
| FurCbox_yfr1_dw | GAATAATTTAAAGTTGGTAATC |  |
| Ifpkn22_up | AAAGATGAATTACACTGGCG | EMSA unspecific control, internal fragment of *pkn22* |
| Ifpkn22_dw | CTGCAAACTGTGGCAGAATA |  |
| PhetZ_dw | GCGTTTAGTTTATCCGCAAA | Promoter region of *alr0099 (hetZ)* |
| PhetZ_dw | CTCAAGCATTGTTGTAGCCG |  |
| Real Time PCR | | |
| alr0092_fw | CCGGGTTGAACTCACTCCAT | *all0092* (*amiC1*) |
| alr0092_rev | ATTCTTATCTACCCGCAAGGTCAT |  |
| amiC2_fw | TCGCATCGCCCAGAGAA | *all0092 (amiC2*) |
| amiC2_rev | GGTGTGTCCACTCCGGTTTC |  |
| all4999_fw | GTCTCCCTACCAGAGCGTCAA | *all4999* |
| all4999_rev | GCGTTGTAATGGACGGAAAGA |  |
| alr2269_fw | ACTAGGCGAACGAGAATTGTTGT | *alr2269* (*omp85*) |
| alr2269_rev | GGTAAGGATCACCACCAATCCA |  |
| all4499_fw | CAACAGACCCTTTGGAAATGC | *all4499* |
| all4499_rev | CCAAGGGTCAGCGTAGAACTG |  |
| alr4067_fw | TTGATGAAGGCCGATTTGTG | *alr4067* (*lptA*) |
| alr4067_rev | TGCTGCGACTCTTCAATCATATAGA |  |
| alr2887_fw | CCAAGTCCGCTTCCAAGTAGA | *alr2887* (*hgdD*) |
| alr2887_rev | TTGGCAGTTTGCACGTTTTC |  |
| rnpB_for | AGCGGAACTGGTAAAAGACCA | *rnpB* housekeeping |
| rnpB_rev | GAGAGGTACTGGCTCGGTAAAC |  |
